# Microbiome Metabolites and Thyroid Dysfunction

**DOI:** 10.3390/jcm10163609

**Published:** 2021-08-16

**Authors:** Piotr Bargiel, Małgorzata Szczuko, Laura Stachowska, Piotr Prowans, Norbert Czapla, Marta Markowska, Jan Petriczko, Jakub Kledzik, Alicja Jędrzejczyk-Kledzik, Joanna Palma, Paulina Zabielska, Dominika Maciejewska-Markiewicz

**Affiliations:** 1 Department of Plastic, Endocrine and General Surgery, Pomeranian Medical University in Szczecin, Siedlecka 2, 72-010 Police, Poland; bergiel87@gmail.com (P.B.); pprowans@wp.pl (P.P.); norbertczapla@gmail.com (N.C.); maciejkam1@gmail.com (M.M.); jan.petriczko@pum.edu.pl (J.P.); jakub.kledzik95@gmail.com (J.K.); jedrzejczykalicja@gmail.com (A.J.-K.); 2 Department of Human Nutrition and Metabolomics, Pomeranian Medical University in Szczecin, 71-460 Szczecin, Poland; malgorzata.szczuko@pum.edu.pl (M.S.); l.stachowskaa@gmail.com (L.S.); 3 Department of Biochemical Science, Pomeranian Medical University in Szczecin, 71-460 Szczecin, Poland; palma.01.01@gmail.com; 4 Subdepartment of Social Medicine and Public Health, Department of Social Medicine, Pomeranian Medical University in Szczecin, 71-210 Szczecin, Poland; paulina.zabielska@pum.edu.pl

**Keywords:** microbiome, thyroid dysfunction, microbiome metabolites, dysbiosis, thyroxine

## Abstract

Thyroid diseases are common conditions that have a negative impact on the health of all populations. The literature sheds light on the differences in the composition of the intestinal microbiota in patients suffering from thyroid diseases compared to healthy individuals. The microbiome affects the proper functioning of the thyroid gland, and the existence of the gut–thyroid axis is discussed in the context of both thyroid diseases and intestinal dysbiosis. The purpose of this review is to describe associations between the microbiome and its metabolites and thyroid dysfunction. We try to explain the role of the microbiome in the metabolism of thyroid hormones and the impact of thyroid autoimmune diseases. In addition, we raise issues related to the influence of bacterial metabolites, such as short-chain fatty acids or secondary bile acids, in the functioning of the thyroid gland. Last but not least, we explored the interactions between the gut microbiota and therapeutics and supplements typically administered to patients with thyroid diseases.

## 1. Introduction

Thyroid diseases are common conditions that have a negative impact on the health of all populations. The diagnosis of thyroid diseases is based on evidence of structural abnormalities of the gland and of altered secretory function. Hormones secreted by the thyroid gland control the function of the majority of tissues, maintaining the internal balance of the body. Iodine is essential for normal thyroid function as well ashormone production [1]. Iodine deficiency is the main cause of thyroid dysfunction in developing countries. It is estimated that about 30% of the world’s population suffers from a deficiency of this element. In these regions, iodine deficiency leads to the development of hypothyroidism and secondary thyroid hypertrophy [2].

Hypothyroidism is one of the most common endocrine disorders, affecting 5–10% of the global population. People who live in developed countries are more likely to be affected by autoimmune diseases, including Hashimoto’s thyroiditis (HT) and Graves’ disease (GD). HT is the most common cause of primary hypothyroidism though it may also presents with hyperactivity and euthyroidism [3]. GD, on the other hand, is the most common cause of hyperthyroidism. Thyroid diseases are several times more common in women than in men; the elderly and patients after radiation exposure are also more vulnerable. Sometimes, the thyroid gland becomes inflamed. HT is the most common type of inflammation and is classified as chronic autoimmune thyroiditis. Acute thyroiditis is rare, mainly affects children, and has a predominantly bacterial etiology. Subacute thyroiditis, also called De Quervain’s thyroiditis, is granulomatous in nature and is possibly caused by a viral infection [4]. The most common structural disorder of the thyroid gland is hypertrophy, known as goitre. It may be diffused, which means the entire parenchyma is enlarged or nodular. Aspiration biopsy should be considered in all cases of nodular lesions in the thyroid gland to assess the risk of malignancy. Tumors of the thyroid gland are mostly benign. Thyroid cancer accounts for 10% of all thyroid neoplasms and is the most common malignant neoplasm of the endocrine glands. In recent years, a significant increase in the incidence of this type of cancer has been observed all over the world. Thyroid cancer may arise from follicular cells (90% of cases) and thyroid C cells. Papillary and follicular are the most common types of thyroid carcinoma, and anaplastic cancer is characterized by rapid growth and poor prognosis, while medullary cancer characteristically secretes calcitonin [5]. The composition of the intestinal microbiota is predominantly shaped by environmental and, to a lesser extent, genetic factors [6]. It has already been proven that an unbalanced diet and medications—including some over-the-counter remedies—as well as chronic stress can cause alterations in the composition of the gut microbiota [7]. This leads to a loss of barrier integrity with all of its consequences. Scientific literature shows that microbiota homeostasis disorders play a crucial role in disrupting tolerance to autoantigens with the concomitant development of autoimmune disorders such as HT [8]. The purpose of this review is to describe the associations between the microbiome and its metabolites and thyroid dysfunction.

## 2. Microbiome and Thyroid Diseases

Human intestinal microbiota consists of billions of bacteria and, to a lesser extent, archaea, viruses, and fungi, and has recently come to be recognized as a ‘hidden’ organ system conducting trophic, metabolic, and immune functions within the human body [9]. Intestinal bacteria are pioneers of immune training. Their continuous cooperation with the immune system that is associated with the intestinal mucosa, namely the gut-associated lymphoid tissue (GALT), is crucial for immune tolerance to commensals and food antigens, while maintaining efficiency in eliminating potentially harmful factors [10]. Intestinal bacteria co-create the intestinal barrier, which is a physical and functional structure within the gut consisting of microbiota, intestinal epithelium and the blood, lymph, and the nervous and GALT systems in the lamina propria. Intestinal barrier integrity is defined as selective permeability to molecules of a certain size and molecular charge. GALT is activated when the ability of the intestinal barrier to control the transport of antigens to the blood vessels is lost. GALT effector cells and proinflammatory factors produced at that time cause subclinical inflammation, initially in situ only [11]. Immunocompetent cells from the intestine migrate to specific tissues and organs, which might consequently initiate persistent inflammation [12]. 

The literature sheds light on the differences in the composition of intestinal microbiota in patients suffering from thyroid diseases compared to healthy individuals. For instance, a study by Zhao et al. [13] demonstrated that the microbiome of patients with HT was of higher richness and diversity compared to healthy controls. The *Firmicutes*/*Bacteroidetes* ratio, used as an indicator of intestinal eubiosis, was elevated in HT patients. Similar relationships have been observed in metabolic syndrome and functional gastrointestinal disorders, where the participation of intestinal microbiota as a key player in the pathogenesis has already been confirmed [14]. A detailed analysis of the results of the genetic testing of the 16S rRNA gene showed that the abundance of *Blautia*, *Roseburia*, the *Ruminococcus torques* group, *Romboutsia*, *Dorea*, *Fusicatenibacter*, and the *Eubacterium hallii* group increased in HT patients, while *Faecalibacterium*, *Bacteroides*, *Prevotella*, and *Lachnoclostridium* were overrepresented in healthy people. Meanwhile, *Bacteroides* effectively ferment fibre into acetate and propionate [15]. *Faecalibacterium* produces butyrate, which is the primary source of energy for colonocytes as well as an important epigenetic regulator of immune responses [16]. Similarly, *Prevotella* and *Oscillibacter* are able to reduce Th17 polarization and enhance the differentiation of anti-inflammatory of regulatory T cells (Treg) cells within the intestine [17]. This is of paramount importance, as a reduction in these bacteria counts was also observed in autism spectrum disorders, a diagnosis with a well-documented inflammatory origin [18,19]. However, the decrease in their numbers clearly reduces the immune potential, which adversely affects selective transport across the intestinal barrier. With regard to the types of bacteria observed in increased numbers, the results are inconclusive. For example, *Blautia* and *Dorea* are described as preferable or unrelated to inflammatory diseases, and species of these genera increase over the course of liver disease, graft-versus-host disease, or Parkinson’s disease [20]. Moreover, *Ruminococcus torques* has the ability to degrade mucin and is a microbial marker of Crohn’s disease [21]. 

Most importantly, however, the results of the study by Zhao et al. [13] showed that there are associations between the abundance of selected types of bacteria and the diagnostic parameters associated with autoimmune thyroiditis, such as antibodies against thyroid peroxidase (anti-TPO) and thyroglobulin (anti-TG). The abundance of 18 types of bacteria was demonstrated to be positively correlated with anti-TPO and anti-TG, while for six types of bacteria, the correlation was negative. A positive correlation for *Alloprevotella* and a negative correlation for *Fusicatenibacter* with FT4 were also demonstrated. Based on linear discriminant analysis, it was concluded that *Bacteroides*, *Streptococcus*, *Faecalibacterium*, *Fusicatenibacter*, *Prevotella*, *Blautia*, *Eubacterium*, *Ruminococcus*, *Alloprevotella*, and *Roseburia* can serve as biomarkers in the non-invasive monitoring of thyroid health [13].

Similarly, another study [22] found that the microbiota of HT patients was characterised by a higher diversity, as assessed by the Chao index, but not the Shannon and Simpson indexes. Patients had lower counts of bacteria from the *Prevotellaceae* and *Veillonellaceae* families, which are both involved in the induction of regulatory T cells in the intestine. Meanwhile, the abundance of *Parasutterella* and *Escherichia coli*, the latter of which responsible for common infections, e.g., of the urinary tract, and the Shiga toxin-producing toxin *Shigella*, were elevated.

An additional factor linking thyroid diseases and gastrointestinal microbiota is the increased risk of developing the former in people with *Helicobacter pylori* infection. Numerous mechanisms are involved, including molecular mimicry, microbial superantigens, high levels of proinflammatory cytokines such as interferon-γ, cross-reactions of the antibodies produced as a result of *Helicobacter pylori* reactions with human tissue antigens, immune complex formation, and the induction of the expression of major molecules of the tissue compatibility complex on the epithelial cells of the thyroid gland [23]. In a study by Aghili et al. [24], concentrations of the anti-*Helicobacter pylori* IgG antibodies TSH and anti-TPO class were assessed. The results proved that 46.5% of the patients from the study group and 10.8% of the controls tested positive for the presence of anti-HP IgG antibodies. In addition, in HT patients infected with *Helicobacter pylori*, after pharmaceutical eradication, anti-TPO and anti-TG antibody titers significantly decreased [25]. In addition, patients with impaired gastric acid secretion required higher doses of thyroid hormone treatment. This relationship suggests that normal gastric pH is needed for the effective absorption of thyroxine [26].

In light of the above, observations regarding the effect of medications on microbiota composition and its metabolic activity are crucial. What is more, intestinal bacteria are equipped with an enzyme apparatus that is involved in the active biotransformation of xenobiotics. This is a new area of research that is known as pharmacomicrobiomics (http://pharmacomicrobiomics.com, accessed on 30 March 2021). There is a body of evidence showing that the interactions between bacteria and xenobiotics can be direct, such as activation, detoxification, direct binding, but also via indirect mechanisms associated with enterohepatic circulation, changes in the kinetics of enzymatic reactions, or the expression of selected genes and bacteria affecting the effectiveness of medications [27]. For example, bacterial azoreductases participate in the formation of the active form, i.e., aminosalicylic acid, from the inactive sulfasalazine taken by the patient. Another example is digoxin, a cardiac glycoside with a narrow therapeutic window, which can be reduced by the enzymes of the *Eggerthella lenta* species, producing inactive 20R-dihydrodigoxin. It has also been proven that some bacteria, such as the above-mentioned *Helicobacter pylori*, can bind medications, which prevents them from reaching their destination and thus limits their therapeutic efficacy. *Helicobacter*-bound Levodopa cannot cross the blood–brain barrier and therefore does not decarboxylate to dopamine in the central nervous system, which renders it ineffective [28].

In summary, a skewed microbiota structure and function might be linked to thyroid disease phenotype. However, as for the existence of a variety of exposomes influencing the ‘bacterial organ’ and more different methodological approaches, there is an urgent need for further prospective studies on the contribution of the intestinal microbiota and its metabolites on thyroid regulation. To add, studies exploring the functional potential of the gut microbiome, including whole genome sequencing and feacal metabolome, are of great importance to be designed. 

## 3. Microbiota and Thyroid Hormone Metabolism

**Figure 1:**
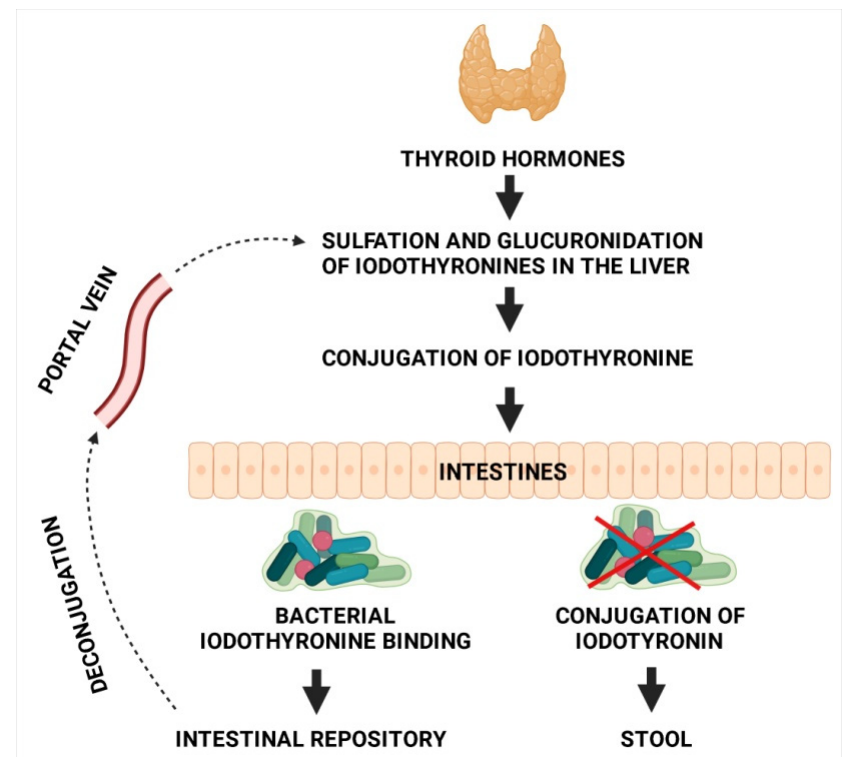
Hepato-intestinal circulation of iodothyronine (created with BioRender.com).

The microbiome affects the enterohepatic cycling of thyroid hormones, the bioavailability of levothyroxine, and the metabolism of propylthiouracil, a medication used in the treatment of hyperthyroidism [29]. In other words, the gut microbiota impacts both endogenous and exogenous thyroid hormones at practically every level. Di Stefano 3rd et al. observed T3 and T4 binding capacity for radioactive iodothyronine in the bacteria comprising the rat intestinal microbiome [30]. According to the study published by Bianco and Kim in 2006 [31], the most effective pathways in the entire iodothyronine metabolism are those of deiodination and conjugation. Deiodinases are distributed asymmetrically in peripheral tissues, ensuring peripheral thyroid homeostasis. Type 2- and 3-deiodinase activities were found to be higher in the intestinal wall of rat foetuses than in adult rats [32]. According to a rat study by Nguyen et al. [33], residual bacteria colonizing the intestine inhibit deiodinase activity. Samples were subjected to a freeze–thaw treatment in order to destroy bacterial cells in the culture. The enzyme activity was at its greatest in the samples prepared in this way. On the other hand, in the presence of the physiological intestinal microbiota enzyme, activity was suppressed, which may be indirectly related to the T3- and T4-binding capacity of the bacteria comprising the microbiota. In a contemporary 2000 study by Sabatino et al. [34], deiodinase activity in the human intestine was confirmed. Conjugation is the next step in iodothyronine metabolism, including the esterification of the phenolic hydroxyl group with sulphuric acid or etherification with glucuronic acid, known, respectively, as sulphoconjugation and glucuroconjugation. The purpose of these reactions is to increase the water solubility of iodothyronine, which on the one hand, facilitates its urinary and biliary clearance, and on the other, reduces its intestinal absorption. To be more specific, sulphoconjugation results in increased levels of inactive metabolites, whereas glucuroconjugation produces considerable amounts of conjugated T4, which are secreted into the intestinal lumen with bile [34]. Intestinal bacteria, especially *Peptococcus productus*, are capable of hydrolysing iodothyronine conjugates, or their deconjugation, thanks to the presence of beta-glucuronidase, whose activity in the intestinal microbiota was demonstrated by de Herder et al. in 1985. In turn, in 1989, Rutgers et al. suggested that gut bacteria are capable of absorbing iodothyronine in the deconjugated form and may therefore serve as a reservoir of the hormone and may even compete with albumins for affinity binding [32]. In one rat study, scholars demonstrated that the intestine is the largest extrathyroidal organ pool of iodothyronine [33]. The hormone may re-enter systemic circulation, thus closing the enterohepatic cycle of iodothyronine. Hepato-intestinal circulation of iodothyronine is shown in Figure 1. 

The intestinal microorganisms co-evolved with the *Homo sapiens*, which emphasizes how many physiological processes are conditioned by their presence. Intestinal microbiota is involved in metabolic, trophic, and immunological functions, and importantly, the products of particular biochemical transformations may serve as substrates of subsequent reactions. From the evolutionary point of view, the most important is the metabolic activity of the microbiota, referred to as the ability to enzymatically decompose nutrients in the digestive tract. However, as presented, the metabolic potential of gut ecosystem also includes thyroid hormones metabolism.

## 4. Mineral Absorption and Microbiome

The process facilitates the uptake of the microelements necessary to ensure the normal metabolism of thyroid hormones, such as iodine, copper, iron, selenium, and zinc [29,35,36]. These minerals are often found to be deficient in patients with thyroid dysfunction. Importantly, these elements are crucial for the thyroid function. For instance, iodine, iron, and copper are pivotal in synthesizing thyroid hormones, while selenium and zinc play a role in T4 to T3 conversion [37].

### 4.1. Iodine

In a rat study conducted in 1972 by Vought et al., gut microbiota was found to affect the intestinal absorption of iodine. Rats were fed kanamycin, an antibiotic effective against both aerobic and anaerobic bacteria commonly found in the lower intestine, especially the Gram-negative *Escherichia coli*. The uptake of radioactive iodine in those rats was lower than in the control group, which was comprised of untreated rats [38]. However, these findings were not corroborated in human studies. In patients with short gut syndrome receiving parenteral nutrition, iodine excretion was at a similar level as in the control group, despite vast disproportions in the presence of gut microbiota between the two groups [39]. Similar conclusions were reached by Michalaki et al. in 2015 in a study investigating urinary iodine excretion levels in patients following bariatric surgery [40]. Even though in light of the above research findings gut microbiota may be presumed to affect the intestinal absorption of iodine, it has no effect on urinary excretion, with the evidence in favour of such a finding being too weak to draw any definitive conclusions [41]. 

### 4.2. Iron

A recent in vitro study showed that *Lactobacillus fermentum*, which is part of the human microbiota, exhibits ferric-reducing activity due to the excretion of p-hydroxyphenyllactic acid, consequently facilitating iron absorption. Moreover, gut microbiota was observed to increase the bioavailability of dietary iron by converting ellagic acid (EA) to urolithin A (UA), which remains active without having to bind Fe^3+^. UA inhibits reactive oxygen species production and protects the host against oxidative stress and inflammatory response [42]. In iron-deficiency states in the body, the expression of divalent metal transporter 1 is upregulated, leading to greater iron absorption and release into the bloodstream, which is mediated by ferroportin—a transmembrane protein found, among others, in duodenal epithelial cells (enterocytes). Likewise, in the case of iron overload, its absorption is downregulated, and the excess iron is incorporated into enterocytes, binding to ferritin—the primary iron-storage protein. Dietary iron deficiency leads to a depletion of intestinal bacteria populations, including *Roseburia*, *Bacteroides,* and *Eubacterium rectale*, and an increase in the *Lactobacillus* and *Enterobacteriaceae* strains [42]. 

### 4.3. Selenium

In food, selenium is found mainly in the form of organic compounds, presumably because it is easier to absorb, and includes the forms of selenomethionine, methylselenocysteine or gamma-glutamyl-methylselenocysteine. However, selenium-enriched foods and dietary supplements, apart from organic selenium compounds, often also contain its inorganic forms, i.e., selenate and selenite. It was demonstrated that the absorption rate for organic selenium compounds is approximately 85–90% of the supplied dose, compared to 10% of the mineral dose supplied in the form of inorganic selenates [42]. Selenium absorption takes place in the duodenum and caecum. Based on findings from animal studies, dietary selenium increases the abundance of bacteria such as *Lactobacillus*, *Bacteroides*, *Prevotella,* and *Roseburia*, while decreasing *Firmicutes*, *Alistipes*, *Parabacteroides*, *Ruminococcus*, and *Helicobacter* [43]. Once absorbed into the bloodstream, selenium binds to plasma albumins and globulins, whereafter it is transported to the liver, kidneys, testes, thyroid, pancreas, pituitary gland, and skeletal muscles. In their 2016 study, Lavu et al. showed in in vitro conditions using a gastric/intestinal absorption model that the selenium absorbed in the small intestine may be actively re-absorbed into the colon and metabolised by the local microbiota, reducing its bioavailability [44]. 

### 4.4. Zinc

Zinc is the second most abundant trace metal in the human body after iron. In 1980, Sandström and Cederblad revealed a trend whereby the higher the zinc content in a meal, the less the fractional absorption of zinc there is in the intestine. After administering 40 μmol of radiolabelled zinc, a 73% absorption rate was achieved, compared to the 46% absorption rate when 200 μmol was administered [45]. In turn, in 1989, August et al. reported that the intestinal absorption of zinc in elderly individuals is lower than in young adults, independent of dietary intake. Zinc absorption is additionally affected by the presence in the intestinal lumen of phytates and other minerals (iron and calcium), which may act as inhibitors binding zinc and blocking its action [46]. In a study with an animal model, elevated serum zinc concentrations were shown in hens supplemented with *Enterococcus faecium* [47]. In another study, an increase in the microbial communities of *Proteobacteria*, coupled with a decline in *Firmicutes* was found in response to zinc therapy in chicks. It was also observed that acute zinc deficiency does not affect the biodiversity of the intestinal microbiome [48].

### 4.5. Copper

There are limited data on copper deficiency and intestinal microbiota composition. Dai et al. studied the effects of early life exposure to copper on the toxicity of gut microbiota in Sprague Dawley rats. The study proved that copper toxicity was dose-dependent and reduced the ratio of *Firmicutes* to *Bacteroidetes.* Additionally, the intervention led to fat metabolism and intestinal inflammation-related bacteria alterations, underlining the negative impact of copper on liver metabolism and intestinal inflammation-related metabolic pathways [49].

In summary, the data exist to prove that the microbiota affects the bioavailability of minerals that are important for the proper metabolism of the thyroid gland.

## 5. The Impact of Oral Thyroid Hormone Supplementation on Microbiome

Oral preparations of thyroid hormones are commonly used worldwide in the treatment of hypothyroidism. They are taken in the morning in a fasting state, with the dosage being dependent on body weight. Oral levothyroxine must cross the intestinal barrier to get into systemic circulation. Intestinal microbiota appears to modulate the expression of tight junctions, affecting intestinal permeability as well as the shape of enterocytes and the composition of the mucus layer, an essential part of the barrier [41]. Animal studies show that germ-free mice have a reduced surface for intestinal absorption, chiefly due to reduced villus height and crypt depth and compromised permeability with impaired transport of macroelements and ions as well as a thinner mucus layer, which also affects the biodiversity of medications. Some gastrointestinal disorders alter microbiome composition, contributing to an increased requirement for oral levothyroxine [50]. 

While there is no conclusive evidence on the role of the microbiome in the malabsorption of oral thyroid hormones, Virili et al. [51] and Cellini et al. [52] observed increased requirements for L-thyroxine in patients with untreated celiac disease and gastrointestinal problems. In a 2017 retrospective cohort study, Brechmann et al. examined the effects of several different factors, including oral levothyroxine replacement therapy, on the development of small intestinal bacterial overgrowth (SIBO). They found that hypothyroidism and L-thyroxine use were the strongest contributors to SIBO [53]. In another study, Yao et al. investigated the relationships between intestinal microbiota and L-thyroxine in patients with subclinical hypothyroidism. 

Research samples from 117 patients were grouped by lipid profile and were categorised into two separate subgroups: patients receiving oral L-thyroxine and patients with no treatment. Patients receiving oral thyroid hormone replacement therapy were additionally subdivided into three small subgroups and treated with low, medium, and high doses of L-thyroxine, respectively. There were discrepancies in the relative abundance of the genera *Odoribacter* and *Enterococcus* depending on the dosage of L-thyroxine, with the greatest abundance observed in the medium dose category, and the lowest abundance shown in those receiving a high dose. Considering the patient group receiving oral thyroid hormone replacement therapy as a whole (irrespective of the dose) versus those with no treatment, the abundance level of bacteria belonging to the genus *Ruminococcus*, which is prevalent in human gut microbiota, was elevated in the group with no treatment. Similar findings were made with regard to the bacteria representing the genera *Alistipes* and *Anaerotruncus* [54]. Researchers from the University of Bradford studied the possible effects of thyroxine on the performance of spatial learning tasks, where cholinergic activity and hippocampal function are of key importance. The control group was made up of rats receiving a saline solution. The experimental group, on the other hand, was given thyroxine at 2.5 or 5 mg/kg/daily over 4 days as a subchronic treatment or at 0.5 or 10 mg/kg administered every third day over 28 days prior to testing as a chronic treatment. The results showed that both the subchronic and chronic treatment with thyroxine significantly improved the ability of rats to learn tasks that required the use of spatial memory, compared to the control group. Moreover, both short-term and long-term thyroxine therapy reduced the effects of scopolamine on cognitive impairment. It was also demonstrated that increased cholinergic activity in the hippocampus and in the frontal cortex was associated with improved performance in the treated animals. These findings point to beneficial effects of thyroxine on cognitive function, possibly mediated by an enhancement of cholinergic activity [55]. Similar observations were made by Fu et al., who administered synthetic levothyroxine to 24-month-old CD-1 mice. The results showed that the levels of choline acetyltransferase, acetylcholine, and superoxide dismutase were increased in the mice from the study group, and their cognitive functions were significantly improved. This suggests that the mechanism by which levothyroxine reverses cognitive impairment is related to choline metabolism [56]. 

Overall, the intestinal bacteria are equipped with an enzyme apparatus involved in the active biotransformation of xenobiotics. There is a body of evidence showing that the interactions between bacteria and xenobiotics can be direct, such as activation, detoxification, direct binding, but also via indirect mechanisms associated with enterohepatic circulation, changes in the kinetics of enzymatic reactions, or the expression of selected genes and bacteria affecting the effectiveness of medications. On the other hand, the results of recently published study showed that it is not only antibiotics that have antimicrobial effect that may result in dysbiosis with all of its consequences. As demonstrated, L-thyroxine therapy has the potential to impact the intestinal health microbiota composition and thus also affect its function. These aspects of pharmacomicrobiomics should be further explored.

## 6. Microbiota and Immune Response in Autoimmune Diseases

Some infections caused by bacteria, their antigens, or viruses are believed to cause autoimmune diseases. For autoimmune thyroid diseases (AITDs), the evidence in favour of this claim is based on retrospective measurements of bacterial antibodies in patients with AITD [57]. In the available literature, there are no clinical trials investigating these issues concurrently with the initial symptoms of infection and ongoing infection, which is obvious when considering the practical challenges. AITDs most commonly involve the presence of anti-TPO, anti-TG, and the thyroid-stimulating hormone receptor (TSHR). It is much more uncommon to detect antibodies against thyroid antigens, such as carbonic anhydrase 2, megalin, T3 and T4, the sodium iodide symporter, and pendrin [28]. The absence of cytotoxicity in GD is one of the main differences with HT [58]. In GD, the inflammatory process mainly affects the thyroid itself but also extends to the adipose tissue, skin, and bones, while the hyperthyroid state impacts the metabolism of the whole body. Interestingly, apart from the characteristic lymphocytic infiltration in HT patients, ultrastructural morphological changes in the enterocytes of the distal duodenum have also been observed, which is potentially indicative of gut dysbiosis [59].

### 6.1. Mechanism Contributing to the Development of AITD in the Course of Infection

The mechanisms leading to autoimmunity following bacterial infection include molecular mimicry, epitope spreading, bystander activation, and the presentation of cryptic antigens [60]. The development of autoimmune thyroid diseases is most often explained by molecular mimicry mechanisms, which is the emergence of autoreactive clones of T and B lymphocytes as a result of a cross-immune response to homologous bacterial or viral antigens [13]. Bacterial infections, which pave the way for molecular mimicry, epitope spreading, and antigen activation, are the key trigger of autoimmune processes.

The group of microorganisms thought to be responsible for the development of autoimmune thyroid diseases includes *Yersinia enterocolitica*, *Helicobacter pylori*, and *Borrelia burgdorferi* as well as *Clostridium botulinum* and *Rickettsia prowazekii* [58,61,62]. Data also suggest a potential pathogenic role of *Toxoplasma gondii*, some strains of *Bifidobacteria* and *Lactobacilli*, *Candida albicans*, and *Treponema pallidum* as well as hepatitis C virus (HCV) [28,58,63]. What is more, some of these microorganisms belong to physiological human microbiota; hence, it may be expected that autoimmune diseases arise as a result of their overgrowth, as in the case of *Candida albicans* following antibiotic treatment. At this stage, however, this is more of a hypothesis than an established scientific fact. For *Borrelia burgdorferi*, shared amino acid sequence homology was demonstrated with human thyroid autoantigens (human thyrotropin receptor (hTSHR), thyroglobulin (hTg), thyroperoxidase (hTPO), sodium iodide symporter (hNIS)), and also between hTSHR and *Yersinia enterocolitica*. The *Borrelia* and *Yersinia* proteins may have the potential to trigger AITDs in individuals with certain HLA-DR alleles [62]. The pathogenesis of both lichen sclerosus (LS) and HT encompasses mutual interaction between genetic and environmental factors. LS is associated with the presence of other autoimmune diseases, including rheumatoid arthritis, systemic lupus erythematosus, pernicious anemia, insulin-dependent diabetes, autoimmune thyroid disease, alopecia areata, vitiligo, and celiac disease. About 40% of LS patients present autoantibodies specific to one or more of the following target organs: thyroid, stomach, parietal cells, or testes; hence, it should be viewed as a multiorgan disease. Several autoimmune polyendocrine syndromes have been diagnosed, with type 3 being the least common, but the pathogenesis of all of them remains unclear [63]. 

### 6.2. Alterations in Gut Microbiome Leading to Augmented Immune Response

The gut microbiome may simultaneously modulate the integration of the immune, metabolic, and endocrine system via the gut–brain axis [64]. Both HT and GD have shown associations with the gut microbiome [22,65]. Gut microbiome analysis in patients with hyperthyroidism shows a significant decline in the populations of *Bifidobacterium* and *Lactobacillus* and an increase in the strains of *Enterococcus*, which may be conducive to dysbiosis [66]. Microbiota influences the enterohepatic cycling of thyroid hormones and the bioavailability of levothyroxine. The altered composition of gut microbiota therefore contributes to the development of AID through the production of autoantigens by the post-translational modification of proteins, the activation of Toll-like receptor 4 (TLR4) induced by lipopolysaccharides (LPS), or the induction of a shift in T helper type 1 cells (Th1) to type 2 (Th2), reducing the integrity of intercellular junctions and inducing transcriptomic, proteomic, and metabolic changes. Toll-like receptors are an element of the innate immune system response to the recognition of virulent pathogens. They play a key role in the production of autoreactive B and T lymphocytes, leading to sensitization and the development of autoimmune diseases [67,68]. Data on the biochemistry and mechanisms initiated by TLR activation may be significant in light of potential therapies for autoimmunity [69,70].

Microbial products, particularly short-chain fatty acids (SCFA), together with thyroid hormones may promote enterocyte differentiation and strengthen intercellular tight junctions, the latter being crucial components of the intestinal barrier, ensuring its integrity [71]. A fecal analysis study showed that four types of intestinal bacteria, i.e., *Veillonella*, *Paraprevotella*, *Neisseria*, and *Rheinheimera*, were capable of distinguishing untreated primary hypothyroidism patients from healthy individuals with the highest accuracy [72]. Patients also present a diminished ability of the normal microbiota to produce short-chain fatty acids, resulting in elevated serum LPS concentrations and interference in endogenous hormone pathways [71]. However, despite the well-documented relationship between microbiota composition and thyroid diseases, the strains that could be useful in supporting therapy still have not been identified. It seems that probiotic therapy may be beneficial for patients with Hashimoto’s thyroiditis accompanied by gastrointestinal complaints, i.e., abdominal pain, bloating, diarrhea, or constipation, or in patients with Hashimoto’s thyroiditis and a concomitant allergy or food intolerance, helping to eliminate intestinal dysbiosis [73]. 

In 2020 Cornejo-Pareja et al. investigated the relationship between gut microbiota composition and AITDs. The compositional analysis demonstrated the increase in bacterial richness in HT and a decrease in GD individuals. Compared to healthy donors, in GD patients, the abundance of *Fusobacteriaceae*, *Fusobacterium*, and *Sutterella* decreased while *Victivallaceae* increased [74].

Taken together, the decreased integrity of the intestinal barrier causes the antigens located in the intestinal lumen to undergo translocation to activate GALT. The GALT effector cells are produced at that time, including the immunocompetent ones as well as pro-inflammatory the factors that elicit subclinical inflammation, which is restricted to the gastrointestinal tract only at the beginning. The presented data support the evidence that bacterial alterations might be risk factors for AITDs development/progression.

## 7. Short-Chain Fatty Acids

When discussing the concept of the gut–thyroid axis, it is worthwhile to mention SCFAs. They are a product of anaerobic fermentation by the gut microbiota of indigestible carbohydrates, notably resistant starch found, e.g., in vegetables and seeds [75]. SCFAs play an essential role in the communication between the gastrointestinal tract and the body as a whole, engaging directly or indirectly in the processes taking part throughout the body. The most important SCFAs are acetic, propionic, and butyric acid, which are present in the intestine in a ratio of 60:25:15, respectively. Their action includes primarily anti-inflammatory mechanisms, the induction of epithelial regeneration processes, and supporting colonic function as well as the indirect modulation of lipid, hormone, and energy metabolism [75,76]. 

It is presumed that the presence of SCFAs may be linked to several different aspects of thyroid function even though the exact processes have not yet been described. From the point of view of the thyroid, it is worth noting the SCFAs’ control over intestinal barrier integrity and their regenerative effects on the intestinal epithelium [77]. SCFAs are a valuable source of nutrients for enterocytes, additionally stimulating their differentiation, together with thyroid hormones (chiefly triiodothyronine) [78,79]. Short-chain fatty acids strengthen intercellular integrity, minimising the risk of a ‘leaky gut’. By improving the adhesion of intestinal cells and reducing pH in the intestinal lumen, SCFAs additionally provide protection against the invasion of pathological organisms [77]. GD and HT are autoimmune disorders, and it has been suggested that their development is often related to a compromised intestinal barrier, enabling pathogenic microorganisms to pass through [37]. As previously mentioned, SCFAs modulate the immune response and have anti-inflammatory properties. In this context, the presence of butyrate is particularly valuable. There is a documented link between its elevated levels and an increased population of Treg in the colon [80]. Treg lymphocytes are responsible for suppressing immune responses that are overly severe or auto-reactive [81]. Additionally, butyrate has also been associated with a reduction in proinflammatory factors [82]. In a very recent study, SCFAs were demonstrated to be able to regulate innate lymphoid cell response not only in the intestines, but also—depending on host condition—in remote tissues [83]. 

The link between SCFAs and thyroid function appears to be confirmed by several reports in the scientific literature describing changes in the gut microbiota, including concentrations of short-chain fatty acids in impaired thyroid status [57,84,85]. In their experiment, Liu et al. compared the gut microbiota in euthyroid and hypothyroid patients [85], and based on the observed differences, they hypothesised that *Phascolarctobacterium* may be involved in the development of HT. What makes this finding significant is the fact that bacteria such as *Phascolarctobacterium feacium* can produce propionate and acetate. In a study on the effects of thyroid dysfunction on the concentrations of acetate, butyrate, and propionate, Dobrowolska-Iwanek et al. observed that impaired thyroid function resulted in reduced levels of the studied substances in rats compared to the control group [84]. Reduced SCFA levels may be the effect of smaller amounts of substrates needed for their production in the colon as a result of delayed intestinal transit in the course of hypothyroidism. 

Research findings suggest that thyroid dysfunction may indirectly affect the concentrations of short-chain fatty acids, while on the other hand, the thyroid was observed to be sensitive to changes in the intestinal microbiota. Additionally, alterations within the composition of the intestinal microbiota result in abnormal metabolic functions of this organ which consequently affect SCFA synthesis. Therefore, further research is necessary to explain the phenomenon of mutual interactions on the thyroid–gut microbiota axis, including the role of SCFAs.

## 8. Secondary Bile Acids

It is widely known that primary bile acids, the most important of which are cholic acid (CA) and chenodeoxycholic acid (CDCA), are produced in the liver as a result of cholesterol metabolism via cytochrome P450 [86]. They are then secreted into the bile and participate in digestion in the form of bile salts. More than 95% is reabsorbed in the ileum, while the remaining close to 5% gets into the colon. There, thanks to the activity of the gut microbiota, they are converted by deconjugation and dehydroxylation into secondary bile acids, which include deoxycholic acid (DCA), lithocholic acid (LCA), and ursodeoxycholic acid (UDCA) [87,88,89]. The microorganisms responsible for the conversion to secondary bile acids represent, among others, the genera *Bacteroides*, *Eubacterium*, *Bifidobacterium*, *Ruminococcus*, and *Clostridia*, with the latter being the most active [29]. 

Secondary bile acids appear to be a potentially powerful instrument modulating systemic homeostasis. This is because they are involved in processes regulating energy metabolism as well as exerting endocrine effects that impact TSH levels [79,89]. Their effects can mainly be attributed to the interaction with two valuable receptors, namely the farnesoid X receptor (FXR) and the G-coupled protein receptor that is specific for the bile acid (TGR5) receptor [90]. TGR5 modulates energy homeostasis, e.g., by influencing insulin sensitivity [88]. It is responsible for activating type 2 iodothyronine deiodinase (D2), which catalyses the conversion of T4 to T3 [91]. FXR, on the other hand, regulates the enterohepatic circulation of bile acids and their biosynthesis via interaction with CYP7A1 [92]. It is therefore a self-regulation mechanism, making sure the synthesis of these acids remains in balance. It needs to be emphasised, however, that bile acid homeostasis is mainly controlled by thyroid hormones, which also affect hepatic CYP7A1, regulate the rate of bile acid synthesis, and may additionally increase their outflow in the liver and gut [79,93]. Moreover, under clinically stable conditions, thyroid hormones may control the breakdown of cholesterol while modulating the primary and secondary bile acids. Reduced levels of low density (LDL) cholesterol are observed in hyperthyroidism, while hypothyroidism is accompanied by its increase [94]. What is more, literature data point to differences in the levels of both the primary and secondary bile acids in patients with impaired thyroid function [95]. In their study, Song et al. demonstrated that patients with subclinical hypothyroidism present reduced serum levels of bile acids [93]. Qi et al. reported that the most prominent secondary bile acid in hypothyroidism is DCA, while CDCA is the most prominent in hyperthyroid patients [29,96]. Liu et al. documented a significant decline in chenodeoxycholic, glycodeoxycholic (GCDA), and deoxycholic acid in hyperthyroid patients, with elevated cholic acid and reduced GCDA in hypothyroid individuals [95]. It was also demonstrated that thyroid hormone lowers LDL cholesterol with a simultaneous suppression of proprotein convertase subtilisin/kexin type 9 (PCSK9) [97]. Moreover, it is hypothesised that one of the reasons for secondary bile acid synthesis, especially UDCA, is their antimicrobial action, protecting the intestines against bacterial overgrowth. A pilot study by Kim et al. showed that the acid reduced the symptoms of SIBO-induced functional dyspepsia in hypothyroid patients [87]. According to the literature data, SIBO often accompanies hypothyroidism. Presumably, this may explain the increased levels of secondary bile acids in hypothyroid patients [98]. 

To summarize, evidence exists to prove that a thyroid hormone imbalance results in the disturbance of secondary bile acid metabolism

## 9. Conclusions

**Figure 2:**
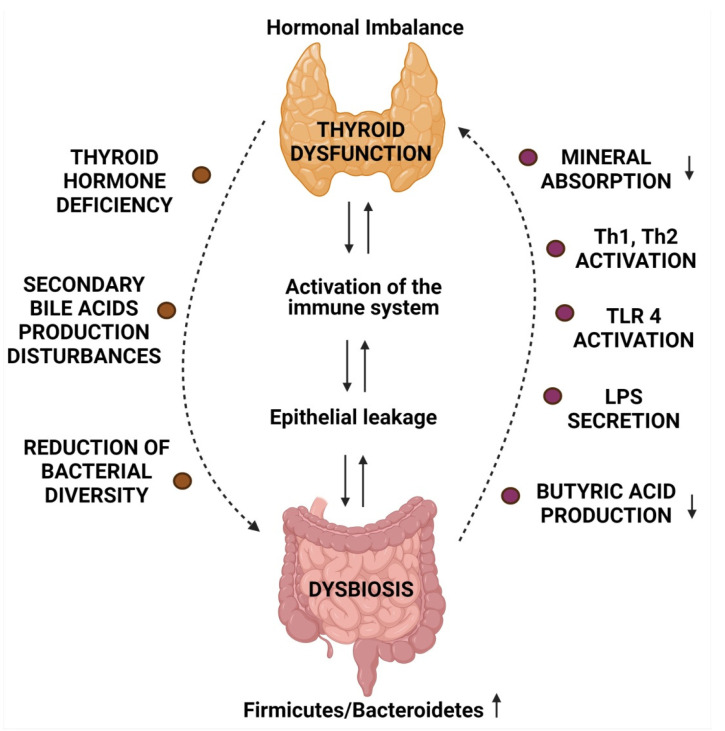
The relationship between dysbiosis and thyroid dysfunction (created with BioRender.com).

The microbiome affects the proper functioning of the thyroid gland, and the existence of the gut–thyroid axis is discussed in the context of both thyroid diseases and intestinal dysbiosis. It is difficult to assess whether dysbiosis is the cause or the effect of thyroid dysfunction, but it is known that the gut microbiome and its metabolites affect thyroid function at many levels. Figure 2 summarizes the relationship between dysbiosis and thyroid dysfunction.

## References

[1] Cuan-Baltazar Y., Soto-Vega E. (2020). Microorganisms associated to thyroid autoimmunity. Autoimmun. Rev..

[2] Vanderpump M.P.J. (2011). The epidemiology of thyroid disease. Br. Med. Bull..

[3] Weetman A., DeGroot L.J., Feingold K.R., Anawalt B., Boyce A., Chrousos G., de Herder W.W., Dhatariya K., Dungan K., Grossman A., Hershman J.M., Hofland J. (2000). Autoimmunity to the Thyroid Gland. Endotext.

[4] Ralli M., Angeletti D., Fiore M., D’Aguanno V., Lambiase A., Artico M., de Vincentiis M., Greco A. (2020). Hashimoto’s thyroiditis: An update on pathogenic mechanisms, diagnostic protocols, therapeutic strategies, and potential malignant transformation. Autoimmun. Rev..

[5] Daly M.C., Paquette I.M. (2019). Surveillance, Epidemiology, and End Results (SEER) and SEER-Medicare Databases: Use in Clinical Research for Improving Colorectal Cancer Outcomes. Clin. Colon Rectal Surg..

[6] Rothschild D., Weissbrod O., Barkan E., Kurilshikov A., Korem T., Zeevi D., Costea P.I., Godneva A., Kalka I.N., Bar N. (2018). Environment dominates over host genetics in shaping human gut microbiota. Nature.

[7] Marlicz W., Yung D.E., Skonieczna-Żydecka K., Loniewski I., van Hemert S., Loniewska B., Koulaouzidis A. (2017). From clinical uncertainties to precision medicine: The emerging role of the gut barrier and microbiome in small bowel functional diseases. Expert Rev. Gastroenterol. Hepatol..

[8] Mori K., Nakagawa Y., Ozaki H. (2012). Does the gut microbiota trigger Hashimoto’s thyroiditis?. Discov. Med..

[9] Heintz-Buschart A., Wilmes P. (2018). Human Gut Microbiome: Function Matters. Trends Microbiol..

[10] Zheng D., Liwinski T., Elinav E. (2020). Interaction between microbiota and immunity in health and disease. Cell Res..

[11] Wang G., Huang S., Wang Y., Cai S., Yu H., Liu H., Zeng X., Zhang G., Qiao S. (2019). Bridging intestinal immunity and gut microbiota by metabolites. Cell. Mol. Life Sci..

[12] Levy M., Thaiss C.A., Elinav E. (2016). Metabolites: Messengers between the microbiota and the immune system. Genes Dev..

[13] Zhao F., Feng J., Li J., Zhao L., Liu Y., Chen H., Jin Y., Zhu B., Wei Y. (2018). Alterations of the Gut Microbiota in Hashimoto’s Thyroiditis Patients. Thyroid.

[14] Drossman D.A., Hasler W.L. (2016). Rome IV—Functional GI Disorders: Disorders of Gut-Brain Interaction. Gastroenterology.

[15] El Hage R., Hernandez-Sanabria E., Calatayud Arroyo M., Props R., Van de Wiele T. (2019). Propionate-Producing Consortium Restores Antibiotic-Induced Dysbiosis in a Dynamic in vitro Model of the Human Intestinal Microbial Ecosystem. Front. Microbiol..

[16] Chen J., Vitetta L. (2020). The Role of Butyrate in Attenuating Pathobiont-Induced Hyperinflammation. Immune Netw..

[17] Li J., Sung C.Y.J., Lee N., Ni Y., Pihlajamäki J., Panagiotou G., El-Nezami H. (2016). Probiotics modulated gut microbiota suppresses hepatocellular carcinoma growth in mice. Proc. Natl. Acad. Sci. USA.

[18] Berding K., Donovan S.M. (2018). Diet Can Impact Microbiota Composition in Children With Autism Spectrum Disorder. Front. Neurosci..

[19] Kang D.-W., Park J.G., Ilhan Z.E., Wallstrom G., LaBaer J., Adams J.B., Krajmalnik-Brown R. (2013). Reduced Incidence of Prevotella and Other Fermenters in Intestinal Microflora of Autistic Children. PLoS ONE.

[20] Bajaj J.S., Hylemon P.B., Ridlon J.M., Heuman D.M., Daita K., White M.B., Monteith P., Noble N.A., Sikaroodi M., Gillevet P.M. (2012). Colonic mucosal microbiome differs from stool microbiome in cirrhosis and hepatic encephalopathy and is linked to cognition and inflammation. Am. J. Physiol. Gastrointest. Liver Physiol..

[21] Hoskins L.C., Agustines M., McKee W.B., Boulding E.T., Kriaris M., Niedermeyer G. (1985). Mucin degradation in human colon ecosystems. Isolation and properties of fecal strains that degrade ABH blood group antigens and oligosaccharides from mucin glycoproteins. J. Clin. Investig..

[22] Ishaq H.M., Mohammad I.S., Guo H., Shahzad M., Hou Y.J., Ma C., Naseem Z., Wu X., Shi P., Xu J. (2017). Molecular estimation of alteration in intestinal microbial composition in Hashimoto’s thyroiditis patients. Biomed. Pharmacother..

[23] Smyk D.S., Koutsoumpas A.L., Mytilinaiou M.G., Rigopoulou E.I., Sakkas L.I., Bogdanos D.P. (2014). Helicobacter pylori and autoimmune disease: Cause or bystander. World J. Gastroenterol. WJG.

[24] Aghili R., Jafarzadeh F., Ghorbani R., Khamseh M.E., Salami M.A., Malek M. (2013). The association of Helicobacter pylori infection with Hashimoto’s thyroiditis. Acta Med. Iran..

[25] Bertalot G., Montresor G., Tampieri M., Spasiano A., Pedroni M., Milanesi B., Favret M., Manca N., Negrini R. (2004). Decrease in thyroid autoantibodies after eradication of Helicobacter pylori infection. Clin. Endocrinol..

[26] Hou Y., Sun W., Zhang C., Wang T., Guo X., Wu L., Qin L., Liu T. (2017). Meta-analysis of the correlation between Helicobacter pylori infection and autoimmune thyroid diseases. Oncotarget.

[27] Koppel N., Rekdal V.M., Balskus E.P. (2017). Chemical transformation of xenobiotics by the human gut microbiota. Science.

[28] Carmody R.N., Turnbaugh P.J. (2014). Host-microbial interactions in the metabolism of therapeutic and diet-derived xenobiotics. J. Clin. Investig..

[29] Fröhlich E., Wahl R. (2019). Microbiota and Thyroid Interaction in Health and Disease. Trends Endocrinol. Metab..

[30] DiStefano J.J., de Luze A., Nguyen T.T. (1993). Binding and degradation of 3,5,3′-triiodothyronine and thyroxine by rat intestinal bacteria. Am. J. Physiol. Endocrinol. Metab..

[31] Bianco A.C., Kim B.W. (2006). Deiodinases: Implications of the local control of thyroid hormone action. J. Clin. Investig..

[32] Virili C., Centanni M. (2014). Does microbiota composition affect thyroid homeostasis?. Endocrine.

[33] Nguyen T.T., DiStefano J.J., Yamada H., Yen Y.M. (1993). Steady state organ distribution and metabolism of thyroxine and 3,5,3′-triiodothyronine in intestines, liver, kidneys, blood, and residual carcass of the rat in vivo. Endocrinology.

[34] Sabatino L., Iervasi G., Ferrazzi P., Francesconi D., Chopra I.J. (2000). A study of iodothyronine 5′-monodeiodinase activities in normal and pathological tissues in man and their comparison with activities in rat tissues. Life Sci..

[35] Stuss M., Michalska-Kasiczak M., Sewerynek E. (2017). The role of selenium in thyroid gland pathophysiology. Endokrynol. Pol..

[36] Triggiani V., Tafaro E., Giagulli V.A., Sabbà C., Resta F., Licchelli B., Guastamacchia E. (2009). Role of iodine, selenium and other micronutrients in thyroid function and disorders. Endocr. Metab. Immune Disord. Drug Targets.

[37] Knezevic J., Starchl C., Tmava Berisha A., Amrein K. (2020). Thyroid-Gut-Axis: How Does the Microbiota Influence Thyroid Function?. Nutrients.

[38] Vought R.L., Brown F.A., Sibinovic K.H., Mc Daniel E.G. (1972). Effect of Changing Intestinal Bacterial Flora on Thyroid Function in the Rat. Horm. Metab. Res..

[39] Navarro A.M., Suen V.M.M., Souza I.M., De Oliveira J.E.D., Marchini J.S. (2005). Patients with severe bowel malabsorption do not have changes in iodine status. Nutrition.

[40] Michalaki M., Volonakis S., Mamali I., Kalfarentzos F., Vagenakis A.G., Markou K.B. (2014). Dietary iodine absorption is not influenced by malabsorptive bariatric surgery. Obes. Surg..

[41] Virili C., Centanni M. (2017). “With a little help from my friends”-The role of microbiota in thyroid hormone metabolism and enterohepatic recycling. Mol. Cell. Endocrinol..

[42] Skrypnik K., Suliburska J. (2018). Association between the gut microbiota and mineral metabolism. J. Sci. Food Agric..

[43] Ren G., Yu M., Li K., Hu Y., Wang Y., Xu X., Qu J. (2016). Seleno-lentinan prevents chronic pancreatitis development and modulates gut microbiota in mice. J. Funct. Foods.

[44] Lavu R.V.S., Van De Wiele T., Pratti V.L., Tack F., Du Laing G. (2016). Selenium bioaccessibility in stomach, small intestine and colon: Comparison between pure Se compounds, Se-enriched food crops and food supplements. Food Chem..

[45] Sandström B., Cederblad A. (1980). Zinc absorption from composite meals II. Influence of the main protein source. Am. J. Clin. Nutr..

[46] August D., Janghorbani M., Young V.R. (1989). Determination of zinc and copper absorption at three dietary Zn-Cu ratios by using stable isotope methods in young adult and elderly subjects. Am. J. Clin. Nutr..

[47] Iftikhar A., Khaliq T., Khan J., Rahman Z., Rahman S., Anwar H., Javed I., Muzaffar H., Mahmood A. (2015). Efficacy of Vitamins, Probiotics and Protein Supplementation on Serum Health Biomarkers of Molted Male Layer Breeders. Pak. Vet. J..

[48] Reed S., Neuman H., Moscovich S., Glahn R.P., Koren O., Tako E. (2015). Chronic Zinc Deficiency Alters Chick Gut Microbiota Composition and Function. Nutrients.

[49] Dai J., Yang X., Yuan Y., Jia Y., Liu G., Lin N., Xiao H., Zhang L., Chen J. (2020). Toxicity, gut microbiota and metabolome effects after copper exposure during early life in SD rats. Toxicology.

[50] Natividad J.M.M., Verdu E.F. (2013). Modulation of intestinal barrier by intestinal microbiota: Pathological and therapeutic implications. Pharmacol. Res..

[51] Virili C., Bassotti G., Santaguida M.G., Iuorio R., Del Duca S.C., Mercuri V., Picarelli A., Gargiulo P., Gargano L., Centanni M. (2012). Atypical Celiac Disease as Cause of Increased Need for Thyroxine: A Systematic Study. J. Clin. Endocrinol. Metab..

[52] Santaguida M.G., Virili C., Del Duca S.C., Cellini M., Gatto I., Brusca N., De Vito C., Gargano L., Centanni M. (2015). Thyroxine softgel capsule in patients with gastric-related T4 malabsorption. Endocrine.

[53] Brechmann T., Sperlbaum A., Schmiegel W. (2017). Levothyroxine therapy and impaired clearance are the strongest contributors to small intestinal bacterial overgrowth: Results of a retrospective cohort study. World J. Gastroenterol..

[54] Yao Z., Zhao M., Gong Y., Chen W., Wang Q., Fu Y., Guo T., Zhao J., Gao L., Bo T. (2020). Relation of Gut Microbes and L-Thyroxine Through Altered Thyroxine Metabolism in Subclinical Hypothyroidism Subjects. Front. Cell. Infect. Microbiol..

[55] Smith J.W., Evans A.T., Costall B., Smythe J.W. (2002). Thyroid hormones, brain function and cognition: A brief review. Neurosci. Biobehav. Rev..

[56] Fu The Synthetic Thyroid Hormone, Levothyroxine, Protects Cholinergic Neurons in the Hippocampus of Naturally Aged Mice. https://www.nrronline.org/article.asp?issn=1673-5374;year=2014;volume=9;issue=8;spage=864;epage=871;aulast=Fu.

[57] Virili C., Fallahi P., Antonelli A., Benvenga S., Centanni M. (2018). Gut microbiota and Hashimoto’s thyroiditis. Rev. Endocr. Metab. Disord..

[58] Fröhlich E., Wahl R. (2017). Thyroid Autoimmunity: Role of Anti-thyroid Antibodies in Thyroid and Extra-Thyroidal Diseases. Front. Immunol..

[59] Sasso F.C., Carbonara O., Torella R., Mezzogiorno A., Esposito V., Demagistris L., Secondulfo M., Carratu’ R., Iafusco D., Cartenì M. (2004). Ultrastructural changes in enterocytes in subjects with Hashimoto’s thyroiditis. Gut.

[60] Ercolini A.M., Miller S.D. (2009). The role of infections in autoimmune disease. Clin. Exp. Immunol..

[61] Benvenga S., Guarneri F. (2016). Molecular mimicry and autoimmune thyroid disease. Rev. Endocr. Metab. Disord..

[62] Benvenga S., Santarpia L., Trimarchi F., Guarneri F. (2006). Human Thyroid Autoantigens and Proteins of Yersinia and Borrelia Share Amino Acid Sequence Homology That Includes Binding Motifs to HLA-DR Molecules and T-Cell Receptor. Thyroid.

[63] Gobaru M., Ashida K., Yoshinobu S., Nagayama A., Kabashima M., Iwata S., Hasuzawa N., Tsuruta M., Wada N., Nakayama H. (2019). Human Leukocyte Antigen (HLA) Subtype-Dependent Development of Myasthenia Gravis, Type-1 Diabetes Mellitus, and Hashimoto Disease: A Case Report of Autoimmune Polyendocrine Syndrome Type 3. Am. J. Case Rep..

[64] Jašarević E., Morrison K.E., Bale T.L. (2016). Sex differences in the gut microbiome–brain axis across the lifespan. Philos. Trans. R. Soc. B Biol. Sci..

[65] Covelli D., Ludgate M. (2017). The thyroid, the eyes and the gut: A possible connection. J. Endocrinol. Investig..

[66] Zhou L., Li X., Ahmed A., Wu D., Liu L., Qiu J., Yan Y., Jin M., Xin Y. (2014). Gut microbe analysis between hyperthyroid and healthy individuals. Curr. Microbiol..

[67] Hosseini A.M., Majidi J., Baradaran B., Yousefi M. (2015). Toll-Like Receptors in the Pathogenesis of Autoimmune Diseases. Adv. Pharm. Bull..

[68] Wallukat G., Schimke I. (2019). Lethal immunoglobulins: Autoantibodies and sudden cardiac death. Autoimmun. Rev..

[69] Dvornikova K.A., Bystrova E.Y., Platonova O.N., Churilov L.P. (2020). Polymorphism of toll-like receptor genes and autoimmune endocrine diseases. Autoimmun. Rev..

[70] Farrugia M., Baron B. (2017). The Role of Toll-Like Receptors in Autoimmune Diseases through Failure of the Self-Recognition Mechanism. Int. J. Inflamm..

[71] Kunc M., Gabrych A., Witkowski J.M. (2016). Microbiome impact on metabolism and function of sex, thyroid, growth and parathyroid hormones. Acta Biochim. Pol..

[72] Su X., Zhao Y., Li Y., Ma S., Wang Z. (2020). Gut dysbiosis is associated with primary hypothyroidism with interaction on gut-thyroid axis. Clin. Sci..

[73] Dolan K., Finley H., Gasta M., Houseman S. (2018). Managing Hashimoto’s Thyroiditis Through Personalized Care: A Case Report. Altern. Ther. Health Med..

[74] Cornejo-Pareja I., Ruiz-Limón P., Gómez-Pérez A.M., Molina-Vega M., Moreno-Indias I., Tinahones F.J. (2020). Differential Microbial Pattern Description in Subjects with Autoimmune-Based Thyroid Diseases: A Pilot Study. J. Pers. Med..

[75] Kuczyńska B., Wasilewska A., Biczysko M., Banasiewicz T., Drews M. (2011). Krótkołańcuchowe kwasy Tłuszczowe–Mechanizmy działania, potencjalne zastosowania kliniczne oraz zalecenia dietetyczne. Now. Lek..

[76] Kumar J., Rani K., Datt C. (2020). Molecular link between dietary fibre, gut microbiota and health. Mol. Biol. Rep..

[77] Memba R., Duggan S.N., Chonchubhair H.M.N., Griffin O.M., Bashir Y., O’Connor D.B., Murphy A., McMahon J., Volcov Y., Ryan B.M. (2017). The potential role of gut microbiota in pancreatic disease: A systematic review. Pancreatology.

[78] Meng S., Wu J.T., Archer S.Y., Hodin R.A. (1999). Short-chain fatty acids and thyroid hormone interact in regulating enterocyte gene transcription. Surgery.

[79] Cayres L.C.d.F., de Salis L.V.V., Rodrigues G.S.P., Lengert A.V.H., Biondi A.P.C., Sargentini L.D.B., Brisotti J.L., Gomes E., de Oliveira G.L.V. (2021). Detection of Alterations in the Gut Microbiota and Intestinal Permeability in Patients With Hashimoto Thyroiditis. Front. Immunol..

[80] Smith P.M., Howitt M.R., Panikov N., Michaud M., Gallini C.A., Bohlooly-Y M., Glickman J.N., Garrett W.S. (2013). The Microbial Metabolites, Short-Chain Fatty Acids, Regulate Colonic Treg Cell Homeostasis. Science.

[81] Czajkowska A., Szponar B. Short Chain Fatty Acids (SCFA), the Products of Gut Bacteria Metabolism and Their Role in the Host. https://phmd.pl/resources/html/article/details?id=168203&language=en.

[82] Köhling H.L., Plummer S.F., Marchesi J.R., Davidge K.S., Ludgate M. (2017). The microbiota and autoimmunity: Their role in thyroid autoimmune diseases. Clin. Immunol..

[83] Sepahi A., Liu Q., Friesen L., Kim C.H. (2021). Dietary fiber metabolites regulate innate lymphoid cell responses. Mucosal Immunol..

[84] Dobrowolska-Iwanek J., Zagrodzki P., Prochownik E., Jarkiewicz A., Paśko P. (2019). Influence of brassica sprouts on short chain fatty acids concentration in stools of rats with thyroid dysfunction. Acta Pol. Pharm. Drug Res..

[85] Liu S., An Y., Cao B., Sun R., Ke J., Zhao D. (2020). The Composition of Gut Microbiota in Patients Bearing Hashimoto’s Thyroiditis with Euthyroidism and Hypothyroidism. Int. J. Endocrinol..

[86] Skonieczna-Żydecka K., Jakubczyk K., Maciejewska-Markiewicz D., Janda K., Kaźmierczak-Siedlecka K., Kaczmarczyk M., Łoniewski I., Marlicz W. (2020). Gut Biofactory—Neurocompetent Metabolites within the Gastrointestinal Tract. A Scoping Review. Nutrients.

[87] Kim B.-T., Kim K.-M., Kim K.-N. (2020). The Effect of Ursodeoxycholic Acid on Small Intestinal Bacterial Overgrowth in Patients with Functional Dyspepsia: A Pilot Randomized Controlled Trial. Nutrients.

[88] Chiang J.Y.L. (2004). Regulation of bile acid synthesis: Pathways, nuclear receptors, and mechanisms. J. Hepatol..

[89] Majewska K., Szulińska M., Michałowska J., Markuszewski L., Bogdański P. (2017). Flora bakteryjna przewodu pokarmowego a choroby układu sercowo-naczyniowego. Forum Zaburzeń Metab..

[90] Pols T.W., Noriega L.G., Nomura M., Auwerx J., Schoonjans K. (2011). The bile acid membrane receptor TGR5: A valuable metabolic target. Dig. Dis..

[91] Thomas C., Auwerx J., Schoonjans K. (2008). Bile acids and the membrane bile acid receptor TGR5—Connecting nutrition and metabolism. Thyroid.

[92] Sonne D.P. (2021). MECHANISMS IN ENDOCRINOLOGY: FXR signalling: A novel target in metabolic diseases. Eur. J. Endocrinol..

[93] Song Y., Zhao M., Zhang H., Zhang X., Zhao J., Xu J., Gao L. (2016). Thyroid-Stimulating hormone levels are inversely associated with serum total bile acid levels: A Cross-Sectional study. Endocr. Pract..

[94] Rizos C.V., Elisaf M.S., Liberopoulos E.N. (2011). Effects of Thyroid Dysfunction on Lipid Profile. Open Cardiovasc. Med. J..

[95] Liu J., Fu J., Jia Y., Yang N., Li J., Wang G. (2020). Serum metabolomic patterns in patients with autoimmune thyroid disease. Endocr. Pract..

[96] Qi X., Yun C., Pang Y., Qiao J. (2021). The impact of the gut microbiota on the reproductive and metabolic endocrine system. Gut Microbes.

[97] Bonde Y., Breuer O., Lütjohann D., Sjöberg S., Angelin B., Rudling M. (2014). Thyroid hormone reduces PCSK9 and stimulates bile acid synthesis in humans. J. Lipid Res..

[98] Gérard P. (2013). Metabolism of Cholesterol and Bile Acids by the Gut Microbiota. Pathogens.

